# Next generation sequencing of PD-L1 for predicting response to immune checkpoint inhibitors

**DOI:** 10.1186/s40425-018-0489-5

**Published:** 2019-01-24

**Authors:** Jeffrey M. Conroy, Sarabjot Pabla, Mary K. Nesline, Sean T. Glenn, Antonios Papanicolau-Sengos, Blake Burgher, Jonathan Andreas, Vincent Giamo, Yirong Wang, Felicia L. Lenzo, Wiam Bshara, Maya Khalil, Grace K. Dy, Katherine G. Madden, Keisuke Shirai, Konstantin Dragnev, Laura J. Tafe, Jason Zhu, Matthew Labriola, Daniele Marin, Shannon J. McCall, Jeffrey Clarke, Daniel J. George, Tian Zhang, Matthew Zibelman, Pooja Ghatalia, Isabel Araujo-Fernandez, Luis de la Cruz-Merino, Arun Singavi, Ben George, Alexander C. MacKinnon, Jonathan Thompson, Rajbir Singh, Robin Jacob, Deepa Kasuganti, Neel Shah, Roger Day, Lorenzo Galluzzi, Mark Gardner, Carl Morrison

**Affiliations:** 1 OmniSeq, Inc., 700 Ellicott Street, Buffalo, NY 14203 USA; 2 Roswell Park Comprehensive Cancer Center, Elm and Carlton Streets, Buffalo, NY 14263 USA; 3 Dartmouth-Hitchcock Medical Center, Lebanon, NH 03756 USA; 4 Duke University Medical Center, 905 S. Lasalle Street, Durham, NC 27710 USA; 5 Fox Chase Cancer Center, 333 Cottman Ave, Philadelphia, PA 19111 USA; 6 Hospital Universitario Virgen Macarena, 41009 Sevilla, Spain; 7 Medical College of Wisconsin, 8701 W Watertown Plank Rd, Milwaukee, WI 53226 USA; 8 Meharry Medical College, 1005 Dr DB Todd Jr Blvd, Nashville, TN 37208 USA; 9 Community Hospital, Munster, IN 46321 USA; 10 University of Pittsburgh, Pittsburgh, PA 15213 USA; 11 Department of Radiation Oncology, Weill Cornell Medical College, New York, NY 10065 USA; 12 Sandra and Edward Meyer Cancer Center, New York, NY 10065 USA; 13 Université Paris Descartes/Paris V, 75006 Paris, France

**Keywords:** Atezolizumab, Avelumab, cancer immunotherapy, Durvalumab, Nivolumab, Pembrolizumab, PD-L1, Biomarker

## Abstract

**Background:**

PD-L1 immunohistochemistry (IHC) has been traditionally used for predicting clinical responses to immune checkpoint inhibitors (ICIs). However, there are at least 4 different assays and antibodies used for PD-L1 IHC, each developed with a different ICI. We set to test if next generation RNA sequencing (RNA-seq) is a robust method to determine *PD-L1* mRNA expression levels and furthermore, efficacy of predicting response to ICIs as compared to routinely used, standardized IHC procedures.

**Methods:**

A total of 209 cancer patients treated on-label by FDA-approved ICIs, with evaluable responses were assessed for PD-L1 expression by RNA-seq and IHC, based on tumor proportion score (TPS) and immune cell staining (ICS). A subset of serially diluted cases was evaluated for RNA-seq assay performance across a broad range of PD-L1 expression levels.

**Results:**

Assessment of *PD-L1* mRNA levels by RNA-seq demonstrated robust linearity across high and low expression ranges. *PD-L1* mRNA levels assessed by RNA-seq and IHC (TPS and ICS) were highly correlated (*p* < 2e-16). Sub-analyses showed sustained correlation when IHC results were classified as high or low by clinically accepted cut-offs (*p* < 0.01), and results did not differ by tumor type or anti-PD-L1 antibody used. Overall, a combined positive PD-L1 result (≥1% IHC TPS and high *PD-L1* expression by RNA-Seq) was associated with a 2-to-5-fold higher overall response rate (ORR) compared to a double negative result. Standard assessments of sensitivity, specificity, positive predictive value (PPV), and negative predictive value (NPV) showed that a *PD-L1* positive assessment for melanoma samples by RNA-seq had the lowest sensitivity (25%) but the highest PPV (72.7%). Among the three tumor types analyzed in this study, the only non-overlapping confidence interval for predicting response was for “RNA-seq low vs high” in melanoma.

**Conclusions:**

Measurement of *PD-L1* mRNA expression by RNA-seq is comparable to PD-L1 expression by IHC both analytically and clinically in predicting ICI response. RNA-seq has the added advantages of being amenable to standardization and avoidance of interpretation bias. *PD-L1* by RNA-seq needs to be validated in future prospective ICI clinical studies across multiple histologies.

**Electronic supplementary material:**

The online version of this article (10.1186/s40425-018-0489-5) contains supplementary material, which is available to authorized users.

## Background

Five trial-evaluated immunohistochemistry (IHC) assays for the assessment of CD274 (best known as programmed death ligand-1, PD-L1) expression in formalin-fixed paraffin-embedded (FFPE) samples have been developed as companion and complementary diagnostics alongside immune checkpoint inhibitors (ICIs) targeting PD-L1 and its main receptor (programmed cell death 1, PDCD1, best known as PD-1) [1–5]. While these tests measure PD-L1 protein levels, they differ by antibody clone, staining platform, and scoring system. For instance, while assessment of PD-L1 expression in advanced gastric cancer or gastroesophageal junction adenocarcinoma samples by the PD-L1 IHC 22C3 pharmDx assay uses a “combined positive score” or CPS [6], testing metastatic non-small cell lung cancer (NSCLC) patient samples relies on a “tumor proportion score” or TPS [7]. This variability in scoring methods has contributed to confounding results across clinical trials and in clinical practice, leading to uncertainty about the universal value of PD-L1 expression levels as a biomarker across tumor types [8–10]. The “Blueprint PD-L1 IHC Comparability Project” was an effort to compare the concordance of five antibody clones commonly used for assessing PD-L1 expression by IHC [11, 12]. The two-phase study revealed a good concordance for three of the five antibody clones employed, but suggests that interchanging assays and cutoffs would lead to “misclassification” of PD-L1 status for some patients. Furthermore, the use of FFPE archival tumor tissues with non-standardized fixation and storage methods may be a source of unpredictable and unintended results for adequate PD-L1 antigen retrieval, potentially increasing the heterogeneity of IHC intensity, extent and topography of staining. All these factors complicate the use of PD-L1 status as assessed by IHC for predicting patient clinical response to ICIs [13, 14].

RNA-based assays on FFPE tissues are currently used in the clinic to classify or predict recurrence risk in patients affected by various tumor types, These assays include DecisionDX-Melanoma (Castle Biosciences), Prosigna® (Nanostring Technologies), MammaPrint® (Agendia), Afirma® Thyroid FNA Analysis (Veracyte), and OncoType DX® (Genomic Health) [15–19]. Most of these tests are microarray- or quantitative reverse transcription (qRT)-PCR-based assays specific for a small panel of cancer-related genes. Recently, RNA-seq has emerged as powerful tool to evaluate mRNA expression in the clinic [20–23]. The use of highly-specific primers that target stably expressed genes provides a high level of specificity and sensitivity, allowing for the simultaneous measurement of several targets including genes for sample quality control purposes. Gene expression profiling by RNA-seq has minimal input requirements and has the potential to be far more cost-effective than IHC methods given the scalability of next-generation sequencing. Further, strong concordance between platforms, including gene expression microarrays, qRT-PCR and IHC has demonstrated the analytic validity of RNA-seq, even for challenging FFPE tumor samples [24]. By digitally counting target molecules, RNA-seq enables precise transcriptome quantification that provides a continuum measurement across a large dynamic range of expression.

The objective of this study was to compare RNA-seq to IHC for the assessment of PD-L1, at both analytical and clinical levels, with the intent to validate RNA-seq as a predictor of response in 209 patients with multiple tumor types treated with ICIs. To demonstrate the linearity and sensitivity of *PD-L1* RNA-seq as a standalone assay, we tested several tumor samples across multiple dilutions. We then used objective response criteria (RECISTv1.1) to compare measurements of PD-L1 by IHC versus RNA-seq to assess clinical utility.

## Methods

### Patients and clinical data

Eight collaborating institutions obtained approval by their respective institutional review boards (IRBs) to submit existing de-identified specimens and associated clinical data for use in this study. Patients were identified for inclusion of electronic pharmacy records indicated they received at least one dose of checkpoint inhibition therapy in the course of standard care, had adequate pre-treatment FFPE tissue (minimum 10% tumor nuclei, maximum 50% necrosis) collected within 2 years of first dose, were evaluable for response by RECIST v.1.1, and had known overall survival from first dose of checkpoint blockade. A total of 209 patients were included, encompassing renal cell carcinoma (RCC, *n* = 45; 7 responders, 38 non-responders), metastatic cutaneous melanoma (*n* = 76; 32 responders, 44 non-responders), and NSCLC (*n* = 88; 17 responders, 71 non-responders). Our primary clinical endpoint for analysis was objective response rate (ORR), defined as patients with complete responses (CR) or partial responses (PR), and patients with progressive disease (PD) or stable disease (SD) classified as non-responders (Additional file 1: Table S1) [25].

### Immunohistochemical studies

In melanoma samples, PD-L1 expression was assessed using the Dako Omnis platform (Agilent, Santa Clara, CA) and the 28–8 pharmDx antibody (Agilent, Santa Clara, CA), which is the FDA-approved complementary diagnostic for nivolumab. For RCC and NSCLC samples, the 22c3 pharmDx antibody (Agilent, Santa Clara, CA) was employed on Autostainer Link 48 (Agilent, Santa Clara, CA), which is the FDA-approved companion diagnostic for pembrolizumab. Established cutoffs for the diagnostics in each histologic type were used to score PD-L1 IHC tumor proportion score (TPS) and immune cell staining (ICS) as follows: melanoma TPS, 1%, [13] NSCLC TPS, 50 and 1%, [7] RCC TPS, 1%; RCC ICS, 1%.

### RNA-seq profiling

RNA was extracted from each sample following microscopic tissue review by an anatomical pathologist and selection of specimen representing tumor cells and associated microenvironment. Gene expression was evaluated by targeted RNA-seq of 384 immune transcripts using an analytically validated assay [23]. Absolute reads were generated using Torrent Suite’s plugin immuneResponseRNA (v5.2.0.0) and further normalized to yield normalized reads per million (nRPM), using previously described methods [23]. For all 394 genes including *PD-L1*, nRPM values were subsequently ranked (gene expression rank) from 0 to 100 based on expression of these genes in a reference population representing a wide range of gene expressions in various tumor types, as previously described [23]. A subset of samples with varying *PD-L1* expression levels were serially diluted to demonstrate sensitivity and linearity of detection.

### Data analysis

To demonstrate the linearity of *PD-L1* mRNA detection, coefficient of determination (R^2^) was calculated for the absolute reads generated across various library dilutions. To investigate the relationship between *PD-L1* expression by targeted RNA-seq and IHC, IHC TPS and ICS results were categorized as either high or low using the previously described FDA-approved complementary and companion diagnostic scoring guidelines and one-way ANOVA and Tukey honest significant difference (HSD) was performed for all PD-L1 values across all samples. To compare IHC versus RNA-seq for prediction of response, values of TPS ≥1% for melanoma, TPS ≥1% and ≥ 50% for NSCLC, and TPS and ICS ≥1% for RCC were compared to RNA-seq expression interpretations of high (rank ≥75) and not-high (rank < 75), relative to a reference population. To compute sensitivity, specificity, positive predictive value (PPV), negative predictive value (NPV), and accuracy, a positive result was considered as IHC TPS of ≥1% for melanoma, TPS of ≥1% and ≥ 50% for NSCLC, and TPS and ICS ≥1% for RCC, and high value for RNA-seq expression (rank ≥75). A negative result was considered as IHC TPS of < 1% for melanoma, TPS of < 1 and < 50% for NSCLC, and TPS and ICS < 1% for RCC, and a moderate or low value for RNA-seq expression. Logistic regression was then performed to evaluate the prediction of response based on tumor type, IHC result, and RNA-seq result.

## Results

### Linearity of *PD-L1* assessment by RNA-seq

**Fig. 1:**
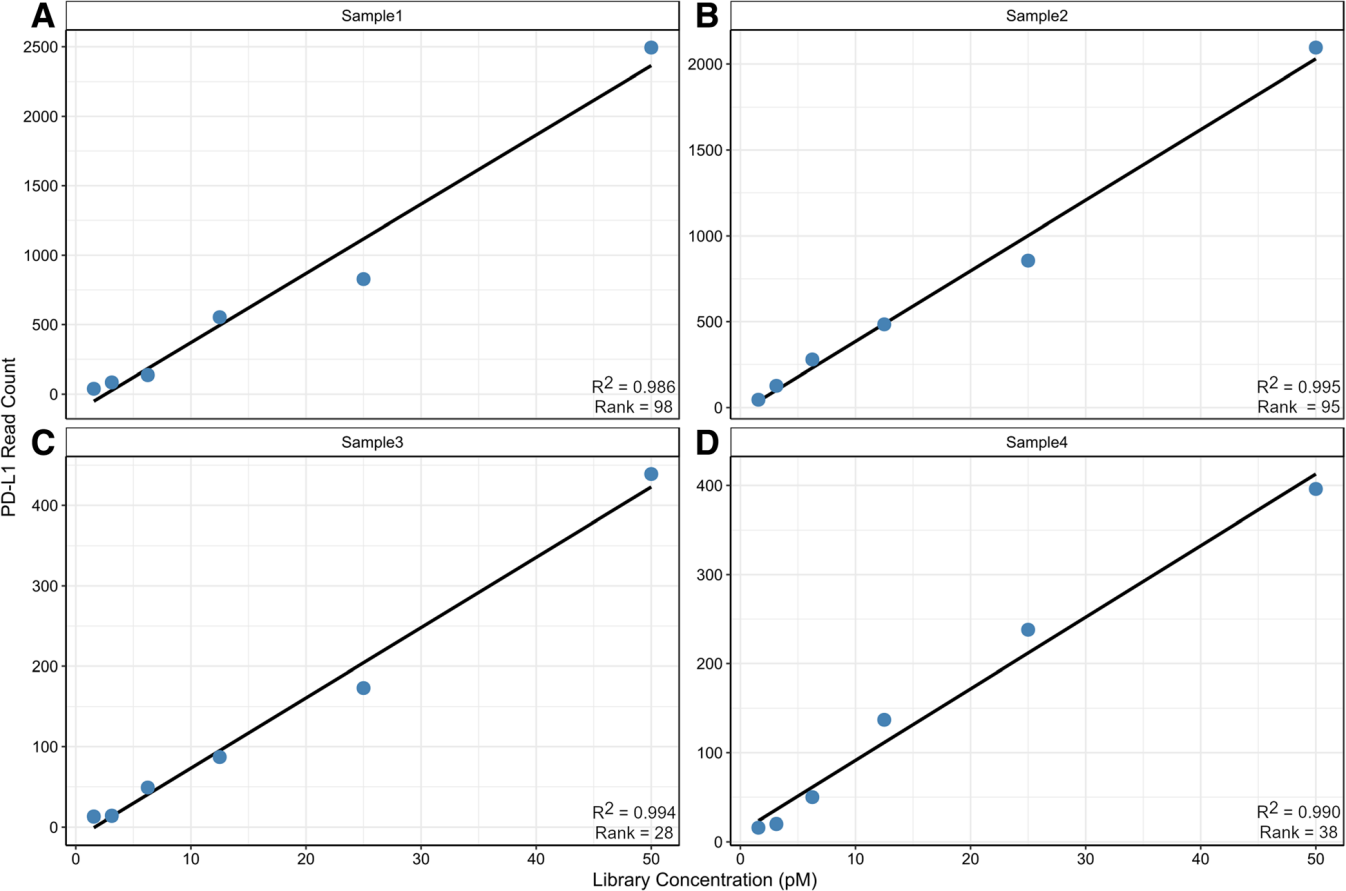
*PD-L1* transcript detection across serial dilutions of 4 tumor samples. *PD-L1* transcript detection across serial dilutions of 4 tumor samples. Results demonstrate high, moderate, and low *PD-L1* expression and can be reliably quantified by a continuous variable of absolute transcript reads. **a** Sample 1: Melanoma with high expression. **b** Sample 2: Melanoma with high expression. **c** Sample 3: RCC with moderate expression. **d** Sample 4: RCC with moderate expression

Linearity of *PD-L1* assessment by RNA-seq was determined by comparing the absolute reads relative to an input of 1.5625, 3.125, 6.25, 12.5, 25, and 50 pM RNA library for tumor samples representing diverse levels of expression (Fig. 1; Additional file 1: Table S2). Samples #1 and #2 represent high expressors (*PD-L1* > 75 rank), while samples #3 and #4 represent moderate expressors (*PD-L1* = 25–75 rank). For samples #1 and #2, *PD-L1* transcript detection values ranged from 0 to > 2400 absolute reads, demonstrating a robust positive linear correlation (R^2^ > 0.98) for clinical specimens expressing high PD-L1 levels. For samples #3 and #4, *PD-L1* transcript detection values ranged from 0 to < 450 absolute reads, demonstrating a positive linear correlation (R^2^ > 0.98) for clinical specimens expressing low-to-moderate PD-L1 levels. Overall, these results demonstrate that detection of *PD-L1* mRNA levels in FFPE samples by RNA-seq is consistent across a dynamic range of expression, and that PD-L1 transcripts can be reliably quantified by a continuous variable of absolute transcript reads down to values approaching background.

### Analytical comparison of IHC and RNA-seq results

**Fig. 2:**
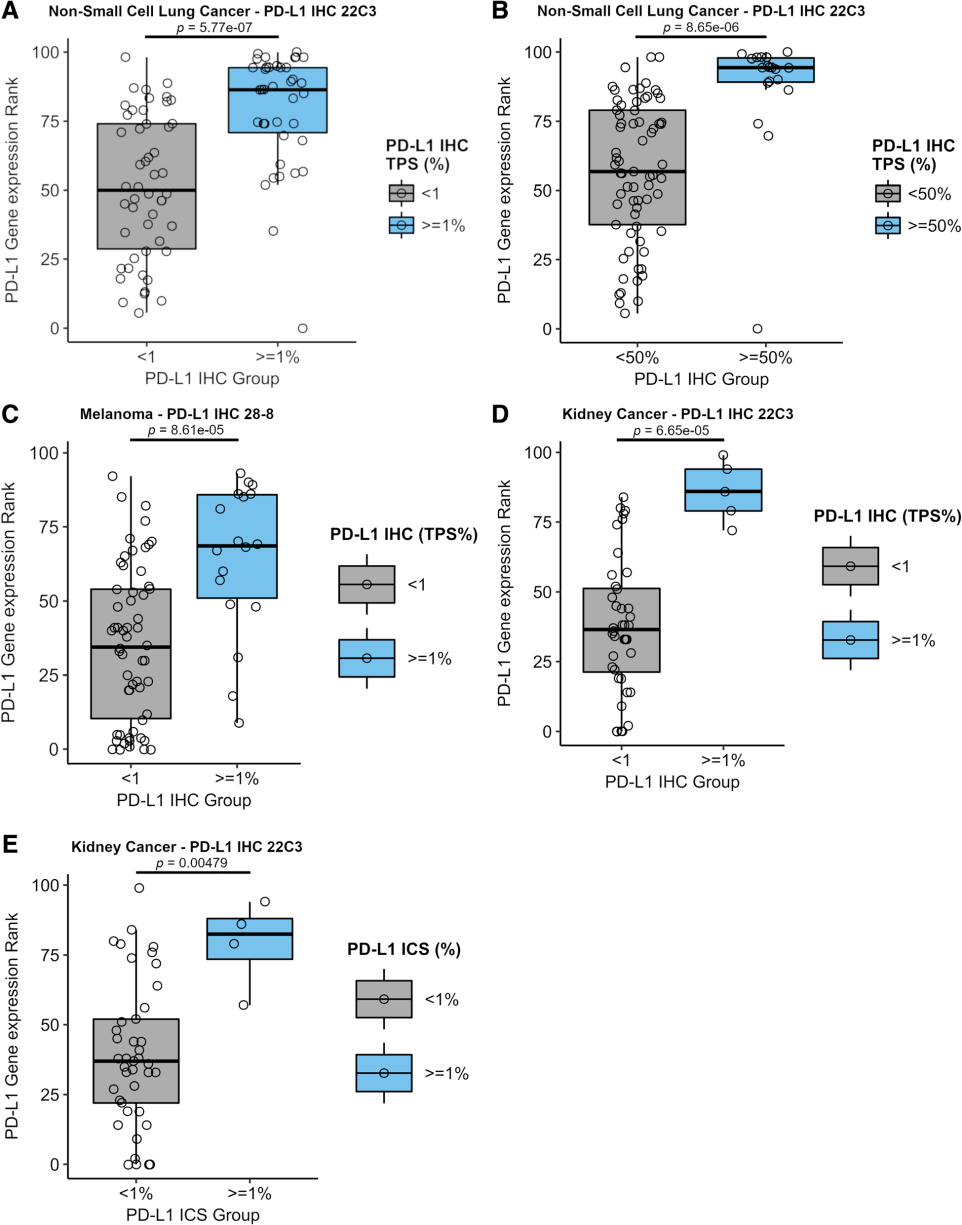
Ad-hoc Tukey’s HSD test comparing *PD-L1* expression by RNA-seq (Y axis) with IHC (X axis). Box plots show concordance of the two measurements across multiple clinical cutoffs for IHC and different tumor types. **a** NSCLC mean TPS at < 1%. **b** NSCLC mean TPS at < 50%. **c** Melanoma TPS mean at < 1%. **d** RCC mean TPS < 1% or E) ICS < 1%

For the 209 samples evaluated, the highest rate of a positive result, defined as IHC TPS of ≥1% for melanoma, TPS of ≥1% and ≥ 50% TPS for NSCLC, and TPS or ICS ≥1% for RCC, or RNA-seq rank ≥75, was observed with NSCLC samples for both IHC and RNA-seq (Table 1). One-way ANOVA demonstrated a statistically significant correlation between PD-L1 RNA-seq rank and IHC (TPS or ICS, *p* < 2e-16) across the three tumor types. The null hypothesis, which specified that the means of *PD-L1* gene expression ranks in each IHC group would not differ, was rejected. Therefore, we performed Tukey HSD for multiple pairwise-comparisons between the means of the IHC TPS/ICS high and not-high groups. Ad-hoc Tukey’s HSD comparisons of NSCLC mean TPS at < 1% (Fig. 2a), NSCLC TPS at < 50% (Fig. 2b), melanoma TPS at < 1% (Fig. 2c), and RCC TPS < 1% (Fig. 2d) or ICS < 1% (Fig. 2e), demonstrated significant differences (*p* < 0.01) between the various groups that were consistent with RNA-seq ranks.

**Table 1:** PD-L1 IHC and RNA-seq results for 209 samples

Test Result	Test	RCC	Melanoma	NSCLC
≥1% TPS	IHC	5 (11%)	19 (25%)	38 (43%)
< 1% TPS	IHC	40 (89%)	57 (75%)	50 (57%)
≥50% TPS	IHC	NA	NA	19 (22%)
< 50% TPS	IHC	NA	NA	69 (78%)
≥1% ICS	IHC	4 (9%)	NA	NA
< 1% ICS	IHC	41 (91%)	NA	NA
> 75 rank (high)	RNA-seq	9 (20%)	11 (14%)	35 (40%)
25–75 rank (moderate)	RNA-seq	24 (53%)	39 (51%)	42 (48%)
> 25 rank (low)	RNA-seq	12 (27%)	26 (35%)	11 (12%)
	Total	45	76	88

### Objective response rates

To investigate the clinical utility of a positive result for PD-L1 by RNA-seq, IHC or both, we evaluated ORR in RCC (*n* = 45), melanoma (*n* = 76), and NSCLC (*n* = 88) patients receiving an FDA-approved ICI (Additional file 1: Table S1). For these comparisons, RNA-seq results ≤75 rank were combined into a single RNA-seq “not high” group, enabling a more direct binary comparison to IHC. The ORR for patients stratified by PD-L1 IHC levels was consistent with previously published values for each tumor type [26–30] (Table 2), supporting that our study population was not biased in selection, and allowing for comparison of the IHC results to those for RNA-seq.

**Table 2:** ORR across tumor type and individual biomarker result

Disease	Test	PD-L1 result	Responders	Non-responders	Total	ORR
Melanoma	IHC TPS	≥1%	10	8	18	55.60%
		< 1%	22	36	58	37.90%
	RNA-seq	High	8	3	11	72.70%
		Moderate & low	24	41	65	46.20%
NSCLC	IHC TPS	≥1%	10	28	38	26.30%
		< 1%	7	43	50	14.00%
	IHC TPS	≥50%	8	11	19	42.10%
		< 50%	9	60	69	13.00%
	RNA-seq	High	10	25	35	28.60%
		Moderate & low	7	46	53	11.90%
RCC	IHC TPS	≥1%	2	3	5	40.00%
		< 1%	5	35	40	12.50%
	IHC ICS	≥1%	1	3	4	25.00%
		< 1%	6	35	41	14.60%
	RNA-seq	High	3	6	9	33.30%
		Moderate & low	4	32	36	8.30%

**Fig. 3:**
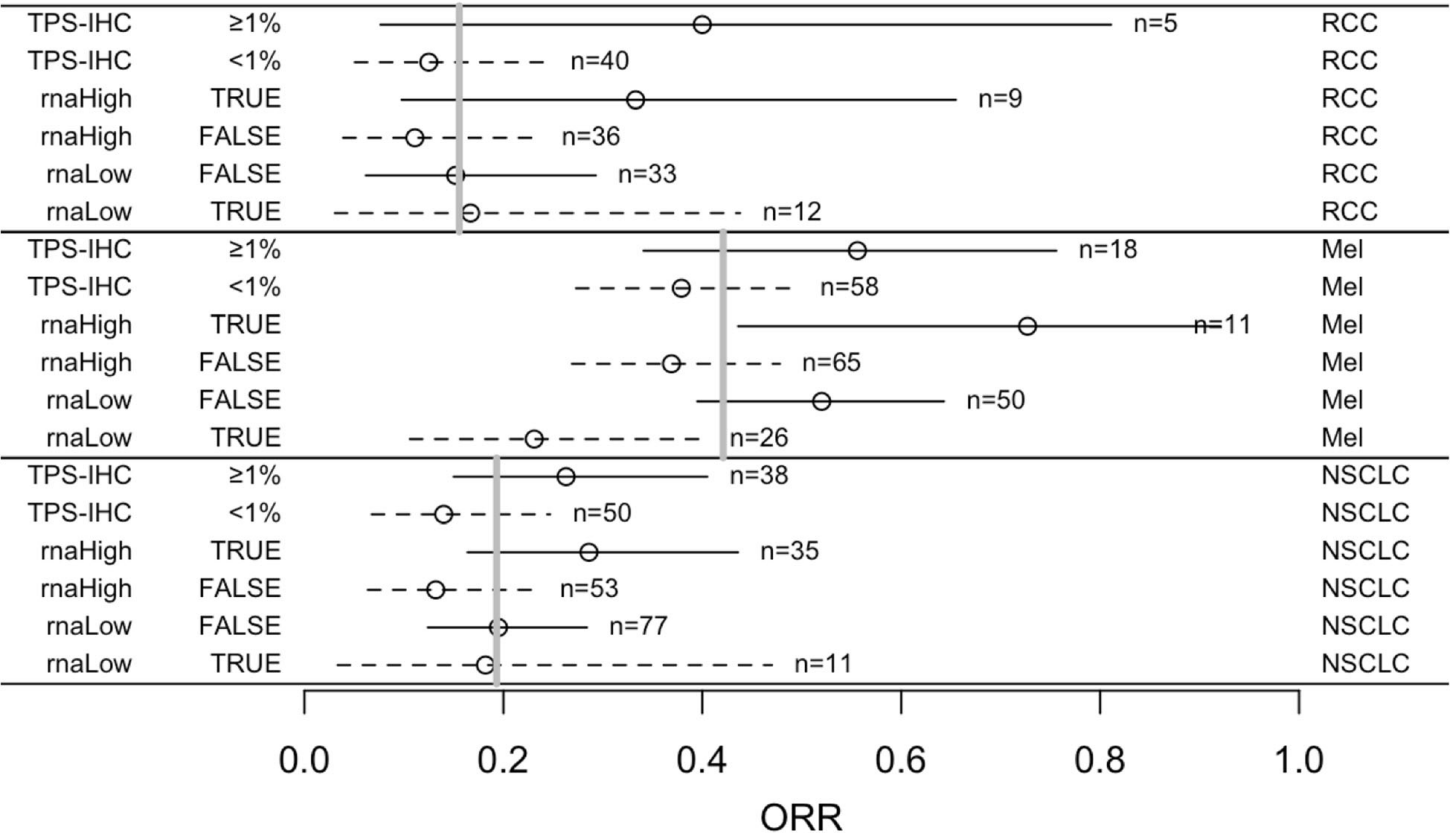
Proportions of responses in subgroups defined by tests for PD-L1 expression. Objective response rate (ORR) was 42.1% for melanoma (Mel), 15.6% for renal cell carcinoma (RCC), and 19.3% for non-small cell lung carcinoma (NSCLC) (grey bars). Each complementary pair of subsets corresponds to positive predictive value (PPV, solid line) and 1 – negative predictive value (NPV, dashed line) (circles). The intervals are 90% confidence intervals. TPS-IHC = PD-L1 tumor proportion score (TPS) by IHC, rnaHigh TRUE = *PD-L1* RNA-seq expression is high, rnaHigh FALSE = *PD-L1* RNA-seq expression is low or moderate (considered “negative”), rnaLow TRUE = *PD-L1* RNA-seq expression is low, rnaLow FALSE = *PD-L1* RNA-seq expression is moderate or high (considered “positive”)

ORR was 42.1% for melanoma, 15.6% for RCC, and 19.3% for NSCLC patients (Fig. 3 and Additional file 1: Table S3). ORR, as measured by various PD-L1 measurements, ranged from 37.9% (IHC < 1% TPS) to 72.7% (RNA-seq high) for melanoma, 11.9% (RNA-seq low) to 42.1% (IHC > 50% TPS) for NSCLC, and 8.3% (RNA-seq low) to 40.0% (IHC > 1% TPS) for RCC. For positive PD-L1 assessments across tumor types, the maximum ORR was 72.7% (RNA-seq) and 55.6% (IHC), 28.6% (RNA-seq) and 42.1% (IHC), and 33.3% (RNA-seq) and 40% (IHC) for melanoma, NSCLC and RCC, respectively. Conversely, negative PD-L1 assessments resulted in ORR of 46.2% (RNA-seq) and 37.9% (IHC) for melanoma, 11.9% (RNA-seq) and 13–14% (IHC) for NSCLC, and 8.3% (RNA-seq) and 12.5–14.6% (IHC) for RCC, with the latter two histologies’ ORR dependent on IHC cutoff and staining interpretation. One important implication of assessing PD-L1 levels is using negative results to support clinical decision making against the administration of ICIs [29]. For RCC, the PPVs for IHC TPS of ≥1% and for RNA-seq high were rather uncertain due to the small population size. In melanoma, only a low RNA-seq result had a notably high NPV (1.0–0.23 = 0.77). For NSCLC samples, neither test had much predictive power for response (Fig. 3).

An important comparison of these two methodologies is the ORR for concordant or discordant results when combining RNA-seq and IHC PD-L1 expression results. Concordant negative results (IHC/RNA-seq −/−) were associated with the lowest observed ORR across all three tumor types. Conversely, concordant positive results (IHC/RNA-seq +/+) were not consistently associated with the highest ORR. Although only four cases, discordant result between IHC and RNA-seq (IHC/RNA-seq −/+) were associated with 75% ORR in melanoma, (3 responders and 1 non-responder), the highest ORR of any tumor. A high ORR was also documented amongst NSCLC patients with discordant results (IHC/RNA-seq +/−) at a ≥ 50% TPS cutoff (ORR = 66.7%), but not at a ≥ 1% value (ORR = 20%) (Additional file 1: Table S4). There were 34 patients treated with ipilimumab, 22 with ipilimumab + nivolumab, and 153 with either single-agent pembrolizumab, nivolumab, or atezolizumab (anti-PD-1). For melanoma, a 67% response rate was observed for patients with RNA-seq high treated with an anti-CTLA4 agent, which increased to 80% when analyzed for anti-PD-1 therapy alone. RCC (*n* = 10) and NSCLC (*n* = 2) had limited number of patients treated with anti-CTLA4 agents, but RNA-seq high was associated with the only RCC response to ipilimumab + nivolumab, as well as non-response for all RNA-seq low patients (Additional file 1: Table S5).

### Clinical utility of PD-L1 IHC versus RNA-seq

Standard parameters of sensitivity, specificity, PPV, NPV, and accuracy were used to compare the clinical utility of PD-L1 assessment with IHC versus RNA-seq (Table 3). RNA-seq in melanoma samples had the lowest sensitivity (25%) and the highest PPV (72.7%) of all test results. The highest sensitivity at 58.8%, shared by both IHC with TPS ≥1% and RNA-seq high in NSCLC samples, was coupled with the lowest PPV at 26.3 and 28.6%, respectively. High NPV (> 85%) was seen with IHC TPS ≥50% and RNA-seq in NSCLC samples, as well as with IHC TPS ≥1% and RNA-seq in RCC samples. However, PPV was suboptimal for all these biomarkers. Sensitivity, specificity, PPV, and NPV for a double positive result (IHC/RNA-seq +/+), as compared to a single positive result, was minimally different from direct comparisons and offered little advantage. The results of these analyses epitomize the typical trade-off of sensitivity for specificity, and vice versa, with an overall less than optimal performance of the tests to predict responders.

**Table 3:** Clinical utility comparison of IHC TPS and RNA-seq rank results

Prediction Method	Sensitivity	Specificity	PPV	NPV
Melanoma IHC ≥1%	31.3%	81.8%	55.6%	62.1%
Melanoma RNA-seq > 75	25.0%	93.2%	72.7%	63.1%
Melanoma IHC ≥1% & RNA-seq > 75	20.8%	94.6%	71.4%	64.8%
NSCLC IHC ≥1%	58.8%	60.6%	26.3%	71.4%
NSCLC IHC ≥50%	47.1%	84.5%	42.1%	87.0%
NSCLC RNA-seq > 75	58.8%	64.8%	28.6%	86.8%
NSCLC IHC ≥1% & RNA-seq > 75	63.6%	68.0%	30.4%	89.5%
NSCLC IHC ≥50% & RNA-seq > 75	46.2%	83.9%	37.5%	88.1%
RCC IHC ≥1%	28.6%	92.1%	40.0%	87.5%
RCC RNA-seq > 75	42.9%	84.2%	33.3%	88.9%
RCC IHC ≥1% & RNA-seq > 75	33.3%	93.9%	50.0%	88.6%

Sensitivity = TP/(TP + FN)

Specificity = TN/(TN + FP)

Positive predictive value (PPV) = TP/(TP + FP)

Negative predictive value (NPV) = TN/(TN + FN)

To evaluate RNA-seq as the gold standard and determine whether IHC adds predictive value, a logistic regression model was employed to evaluate the prediction of response to treatment based on tumor type, PD-L1 by IHC, and *PD-L1* levels by RNA-seq (Table 4). As expected, the model shows that melanoma patients (*p* = 0.0026) have a higher response rate than patients with RCC and NSCLC, and that the expression rank interpretation (“RNA-seq”) has a significant linear (“RNA-seq.L”) relationship to response (equally spaced scoring from low to moderate, and from moderate to high). The RNA-seq.L estimate, 0.96, is the increase in the log odds of response moving from low to moderate, or from moderate to high (odds increased by a factor of 2.6, and by 6.8 going from Low to High). There is no indication of a further quadratic effect (“RNA-seq.Q”), but the sample size is small for detecting such a departure from equal spacing. A model examining whether the relationship between RNA-seq and ORR varies by tumor type showed no significant interaction (*p* = 0.80). A model examining whether the *PD-L1* RNA-seq and IHC results interact in predicting response also showed no significant interaction (*p* = 0.45). Among the three tumor types analyzed in this study, the only non-overlapping confidence interval for predicting response was for “RNA-seq low vs high” in melanoma.

**Table 4:** Logistic regression for predicting response category “CR or PR” versus “SD or “PD”

	Estimate	Std. Error	z value	Pr(>|z|)
TumorType RCC	(reference)	–	–	–
TumorType Melanoma (versus RCC)	1.50	0.50	3.01	0.0026
TumorType NSCLC (versus RCC)	−0.13	0.53	−0.25	NS
IHC TPS ≥1%	0.41	0.40	1.03	NS
RNA-seq.L	0.96	0.40	2.43	0.015
RNA-seq.Q	0.21	0.28	0.76	NS

## Discussion

PD-L1 appears as one of the most controversial biomarkers to be introduced into clinical practice. Despite prior evidence demonstrating that both technologic and histologic variability limit clinical utility [31, 32], four IHC based tests are currently approved for guiding treatment decisions in patients with multiple tumor types. Clinicians, patients and other stakeholders seeking reliable PD-L1 diagnostic assays are subjected to the lack of IHC standardization and face real implications to clinical care. Recently, the Blueprint Working Group was established with cooperation from the FDA, IHC platform vendors, professional oncology organizations and the pharmaceutical industry to provide a comparison of different PD-L1 IHC tests in NSCLC, including scoring methods for expression [11] . The initial findings showed comparable results for 22C3, 28–8, and SP263 antibody clones, but reduced sensitivity for the SP142 clone, when evaluating staining in tumor cells. In the phase 1 study, only 50% of the cases demonstrated concordant positive staining above the antibody specific cutoffs. These results are alarming given that the Blueprint study involved pathologists with considerable expertise in NSCLC and the utilization of large resection specimens (*n* = 38) that are typically easier to evaluate. While ‘no’ and ‘very high’ PD-L1 expression were mostly concordant, the Blueprint study demonstrated that the ‘low’ to ‘intermediate’ expression levels seen in most NSCLC patients can result in discrepancy. In these instances, a continuous measure, rather than using a specific TPS cutoff, may better predict ICI treatment efficacy. The more recent phase 2 study [12] confirms the previously reported differences in sensitivity between the IHC assays, and reports reliability among pathologists ranging from very strong for TPS scoring to poor for immune cell scoring. Given the challenges associated with PD-L1 IHC, it is surprising the paucity of information that exists for PD-L1 measurement in FFPE tumors by other methods.

In contrast to the Blueprint study, our study explores measuring PD-L1 by a single alternative methodology, RNA-seq by direct comparison between FDA-approved PD-L1 IHC assays and a laboratory developed RNA-seq test. Although previous studies demonstrate PD-L1 as measured by IHC is a predictive biomarker of response to ICIs [33], it was unclear if an alternative methodology would validate PD-L1 utility as a predictive biomarker. It was not the intent of this study to debate the clinical utility of PD-L1 IHC, but to assess the clinical utility of *PD-L1* by RNA-seq. Unlike IHC, RNA-seq quantitates the number of expressed mRNA transcripts in the entire tumor microenvironment without subjective scoring methods and cell type discrimination. When performed in a CLIA laboratory setting with a validated protocol [23], our data demonstrates that RNA-seq is a highly sensitive and robust assay for measuring *PD-L1* across a continuum of expression levels.

Our study employed a considerably large (*n* = 209) cohort of samples from multiple institutions treated with one or more FDA-approved ICIs. The specimens were of variable tumor mass, including a large fraction of needle core biopsies and FNA cell blocks. Furthermore, multiple tumor types were evaluated and the pathologist reading IHC slides was not a renowned expert in any one particular disease. In total, the study reflects a real-world clinical scenario in which archival specimens representing several commonly tested histologies are evaluated for PD-L1 expression.

Data obtained from this study revealed that *PD-L1* expression as measured by RNA-seq is highly correlated to IHC both analytically and clinically. Overall, ICI response varied between tumor types but as expected, each demonstrated highest ORR with either a PD-L1 positive RNA-seq or IHC. We acknowledge that the relatively small number of PD-L1 positive cases, especially RCC, may limit the evaluation of RNA-seq as a predictive assay for the tumor types evaluated, but ORR for patients stratified by PD-L1 IHC levels was consistent with previously published values. Even with these limitations however, RNA-seq high and low results for melanoma demonstrates significant PPV and NPV, respectively, and resulted in an overall 73% ORR compared to 56% ORR by PD-L1 IHC. Although the combined positive (IHC/RNA-seq +/+) samples were associated with an overall higher ORR than the combined negative (IHC/RNA-seq −/−) samples, the highest ORR of any tumor type were associated with discordant melanoma IHC and RNA-seq results (IHC/RNA-seq −/+). Given the small number of samples (*n* = 4) in this grouping, a larger cohort with this phenotype is required to understand if the predictive power is tumor type specific or a direct result of *PD-L1* quantitation in melanoma by RNA-seq. NSCLC samples, which had the largest number of PD-L1 positive RNA-seq and IHC cases, did not however demonstrate predictive power for either test. Most interesting is the ORR for combined positive (IHC/RNA-seq +/+) and negative (IHC/RNA-seq −/−) results. While a combined positive did not always demonstrate the highest ORR across tumor types, the combined negative result did have the lowest ORR, except for RCC IHC/RNA-seq +/− which was represented by only one case.

Unexpectedly, we found that melanomas with PD-L1 IHC TPS > 1% had a significantly improved response to all ICI monotherapies, including anti-PD-1 and anti-CLTA-4 monotherapy. We were also surprised to find that elevated *PD-L1* by RNA-seq quantification was an even better predictor of response to the same monotherapies. This is a provocative finding given that the PD-1/PD-L1 axis is currently thought to be entirely distinct and not interacting with the CTLA-4 axis [34]. However, we do acknowledge that our melanoma data set is limited in size and this finding needs to be confirmed in future studies.

In addition to the inherent advantages of standardized methods for *PD-L1* assessment by RNA-seq, this technology is also convenient as it enables highly multiplexed testing of several patients in a single run, with per sample costs approaching those of IHC when performed in batch sizes greater than twenty. By measuring multiple transcripts simultaneously, RNA-seq is well suited to characterize the functional state of immune cells in the tumor microenvironment for biomarkers of antigen presentation, IFN-γ signaling, T-cell active cytokines and other biological features that are responsive to PD-1 checkpoint blockade. It is beyond the scope of this study to report data for the focused set of nearly 400 other genes included in the transcriptome panel, however evaluating RNA for immune gene expression in addition to PD-L1 has been shown to be predictive of efficacy to anti-PD-1 therapy across multiple tumor types with more accuracy than PD-L1 IHC [35–37]. For example, a T-cell inflamed signature based on IFN-γ genes was associated with response to anti-PD-1 therapy in multiple tumor types [35], and an algorithmic approach which combines gene expression profiling with tumor mutational burden (TMB) and PD-L1 IHC improves prediction of response to ICIs in melanoma [37]. These multi-marker approaches contribute more comprehensive information to the cancer immunity cycle [38] than a single analyte and could improve personalized combination immunotherapy treatment options in patients that have failed prior immunotherapy by targeting over-expressed immunomodulatory factors, including LAG-3, GITR, ICOS, TIM-3, and OX40 [39] across multiple tumor types [40, 41]. Co-overexpression of PD-L1 and PD-L2 (another PD-1 ligand) in the same tumor, as well as overexpression of other co-inhibitory or co-activatory molecules can reliably indicate whether checkpoint blockade is a significant factor in a specific case [42, 43]. Though not presented here, RNA-seq may enable a qualitative and quantitative analysis of tumor infiltrating lymphocytes that inform on whether the tumor is immunologically “hot” or “cold”, and provide data to explore new biomarker opportunities [32].

For analytical purposes, the expression of other genes has value relative to PD-L1 for use as an endogenous control for PD-L1 normalization. It is feasible that the various subjective IHC interpretation requirements for estimating percentages of either tumor, immune or combined cell staining could be replaced by normalization of *PD-L1* RNA-seq by cell specific markers. Normalization against one or several markers, such as an immunohistochemical stain which the surgical pathologist determines to be highly specific for neoplastic cells in a given case, CD45 for hematopoietic cells, CD3 for T-cells, CD8 for cytotoxic T-cells, or CD68 for macrophages could result in an objective qualitative PD-L1 result that can be automated to reproducibly report *PD-L1* expression relative to specific cell types in the tumor microenvironment. With proper tissue review and selection, RNA-seq analysis of the tumor and associated microenvironment is designed to provide a PD-L1 score minimally influenced by sample selection bias and tumor heterogeneity, and to minimize the current PD-LI testing variability which may impact clinical decisions and the uptake of precision immune oncology treatments [44].

A major limitation of our study linked to development of RNA-seq as a standardized measurement of *PD-L1* expression, is a lack of external standards. In our study, RNA-seq measurements have a proprietary method of normalization that influence the results for all genes in the panel. Our control samples and genes with a similar impact are also proprietary. Additionally, the interpretation of these measurements by rank is derived via comparison to a unique non-public reference database. Although these are significant issues, the standardization of *PD-L1* measurement by RNA-seq is much more attainable than in the case of IHC. External, publicly available standards could be developed and shared similarly to how minimal residual disease for BCR-ABL has become standardized [45]. Another limitation of this study is its retrospective nature of testing archival specimens to assess the presence of a dynamically upregulated biomarker which can change during the disease course. Therefore, PD-L1 status by RNA-seq needs to be further validated in future prospective ICI clinical trials.

The confluence of the Blueprint study and our work supports that alternative measurements of PD-L1 expression beyond IHC, such as RNA-seq, should be considered for clinical use to improve response prediction in patients being considered to receive ICI treatments.

## Conclusions

In summary, our study shows that measurement of *PD-L1* mRNA expression by RNA-seq is comparable to PD-L1 expression by IHC assays, both analytically and clinically, with evidence that for melanoma samples RNA-seq may be superior to IHC. At minimum, mRNA expression by RNA-seq provides another layer of PD-L1 detection which can exploited to predict tumor response to ICI. The predictive performance of RNA-seq to measure *PD-L1* expression (in comparison to IHC) is affected to some extent by histologic factors, but the overall results suggest that moving forward with this technology is a viable approach for this dynamic biomarker.

## Additional file

Additional file 1: **Table S1.** Samples (*n*=209) sorted within tumor type by PD-L1 rank (RNA-seq). **Table S2.** PD-L1 (CD274) RNA-seq dilution series. **Table S3.** Responders and non-responders across tumor types and biomarker result. **Table S4.** ORR across tumor type and combined biomarker results for all ICI therapies. **Table S5.** Responders and non-responders across tumor types and individual biomarker result. (XLSX 39 kb)
